# Machine Learning-Based Position Detection Using Hall-Effect Sensor Arrays on Resource-Constrained Microcontroller

**DOI:** 10.3390/s25206444

**Published:** 2025-10-18

**Authors:** Zalán Németh, Chan Hwang See, Keng Goh, Arfan Ghani, Simeon Keates, Raed A. Abd-Alhameed

**Affiliations:** 1 School of Computing, Engineering and the Built Environment, Edinburgh Napier University, Edinburgh EH10 5DT, UK; zal.nemeth@gmail.com (Z.N.); k.goh@napier.ac.uk (K.G.); 2 Department of Computer Science and Engineering, American University of Ras al Khaimah, Ras al Khaimah 72603, United Arab Emirates; arfan.ghani@aurak.ac.ae; 3 Centre for Future Technologies, University of Chichester, Chichester PO19 6PE, UK; s.keates@chi.ac.uk; 4 Faculty of Engineering and Digital Technologies, University of Bradford, Bradford BD7 1DP, UK; r.a.a.abd@bradford.ac.uk; 5 Department of Information and Communication Engineering, Al-Farqadein University College, Basrah 651004, Iraq

**Keywords:** machine learning, Hall-effect sensor array, electromagnetic levitation system, microcontroller, TinyML

## Abstract

This paper presents an electromagnetic levitation system that stabilizes a magnetic body using an array of electromagnets controlled by a Hall-effect sensor array and TinyML-based position detection. Departing from conventional optical tracking methods, the proposed design combines finite-element-optimized electromagnets with a microcontroller-optimized neural network that processes sensor data to predict the levitated object’s position with 0.0263–0.0381 mm mean absolute error. The system employs both quantized and full-precision implementations of a supervised multi-output regression model trained on spatially sampled data (40 × 40 × 15 mm volume at 5 mm intervals). Comprehensive benchmarking demonstrates stable operation at 850–1000 Hz control frequencies, matching optical systems’ performance while eliminating their cost and complexity. The integrated solution performs real-time position detection and current calculation entirely on-board, requiring no external tracking devices or high-performance computing. By achieving sub 30 μm accuracy with standard microcontrollers and minimal hardware, this work validates machine learning as a viable alternative to optical position detection in magnetic levitation systems, reducing implementation barriers for research and industrial applications. The complete system design, including electromagnetic array characterization, neural network architecture selection, and real-time implementation challenges, is presented alongside performance comparisons with conventional approaches.

## 1. Introduction

Magnetic levitation (Maglev) technology has garnered widespread recognition for its transformative capabilities in high-precision positioning systems, with applications ranging from industrial automation and transportation to semiconductor manufacturing [1]. The core advantages of Maglev systems include the elimination of mechanical wear, friction, backlash, and vibration, which are prevalent in traditional contact-based systems. These benefits contribute to enhanced operational efficiency, extended system lifespans, and precise control over six degrees of freedom (DOF) with minimal physical interaction. Despite these notable benefits, the widespread adoption of Maglev technology is hindered by challenges such as inherent nonlinear behavior [2,3], limited motion ranges, and the complexities of achieving stable levitation over large distances. Recent advancements in electromagnetic actuation, feedback control mechanisms, and magnetic array designs have begun addressing these limitations, improving system performance [4,5].

Maglev systems have demonstrated the potential to revolutionize traditional processes across various fields. For instance, in space-based optical communication, Maglev technology enables precise pointing mechanisms for accurate data transmission [6]. In automated assembly, it enhances precision and efficiency through magnetically levitated robot arms [7]. The semiconductor industry benefits from high-precision multidimensional positioners that improve fabrication processes [8]. Additionally, Maglev is integral to aerodynamic testing, providing unobtrusive and accurate measurements via magnetic suspension and balance systems [9,10,11]. In haptic systems, Maglev expands motion ranges, delivering more immersive experiences [12].

The fundamental principle of Maglev is based on magnetic repulsion or attraction, where electromagnets or permanent magnets generate fields to counteract gravitational forces. However, stable levitation is inherently challenging due to Earnshaw’s theorem [13], which states that achieving static equilibrium with fixed magnets is impossible. To overcome this limitation, dynamic feedback control systems are employed to adjust magnetic forces in real time, ensuring stability. Maglev systems can be broadly categorized into restricted and non-restricted systems based on their translational ranges. Restricted systems, such as Maglev trains, operate within predefined spatial domains to ensure objects remain confined [5,14]. Conversely, non-restricted systems (the focus of this paper) provide greater flexibility, allowing levitated objects to move freely within operational bounds making them essential for applications demanding spatial versatility.

Over the years, significant progress has been made in non-restricted Maglev systems. In 2007, Lai, Lee, and Yen [15] developed a 6-DOF electromagnetic levitation system utilizing a combination of solenoids and permanent magnets, stabilized with a proportional–integral–derivative (PID) controller. Berkelman and Dzadovsky [13] extended this work by implementing an electromagnetic planar array (EMPA) with proportional–derivative (PD) control, later enhancing it to a 16-coil array for broader translational ranges [16]. In 2014, they introduced a device capable of unlimited omnidirectional rotation [17]. In 2020, Mousa Lahdo et al. [18] proposed a decoupled levitation and propulsion system using eddy current and laser sensors, while Berkelman and Yu-Sheng [19] developed a cost-effective photodiode-based position detection method. More recently, by using gap sensor and current sensor, an optimization algorithm for the real-time levitation performance optimization under the implementation structure was developed [4] in 2023, while in 2024, [20] employed the gap self-sensing method using partial electromagnetic coil could obtain accuracy gap information in time to achieve stable levitation.

Building on these advancements, this paper introduces a novel Maglev system that integrates Hall-effect sensors with machine learning (ML) techniques. Specifically, the system employs Tiny Machine Learning (TinyML)—a subset of ML optimized for resource-constrained microcontroller environments. By leveraging TinyML, the proposed system achieves efficient inference reducing complexity and cost while maintaining high performance [21,22,23]. Beyond accurate position detection, the framework is embedded directly into a microcontroller, enabling on-board real-time computation of both position and coil currents without reliance on external tracking devices or high-performance processors. This integration ensures control frequencies approaching 1 kHz required for stable levitation, while retaining the scalability and portability necessary for wider industrial and research applications.

The contributions of this work are summarized as follows:

1. A detailed design and implementation of a Hall-effect sensor-based position detection system integrated with TinyML on microcontrollers.
2. Development of a quantized and non-quantized machine learning algorithm optimized for microcontroller deployment.
3. Experimental validation demonstrating high accuracy, low latency, and robust performance across diverse scenarios.

This paper is organized as follows: Section 2 provides an overview of the proposed system describing the high-level operation, Section 3 outlines the hardware design, and Section 4 presents software development. The experimental results and their analysis are discussed in Section 5 detailing system limitations and future research directions and finally, conclusions are presented in Section 6.

## 2. Overview of the Proposed System

**Figure 1:**
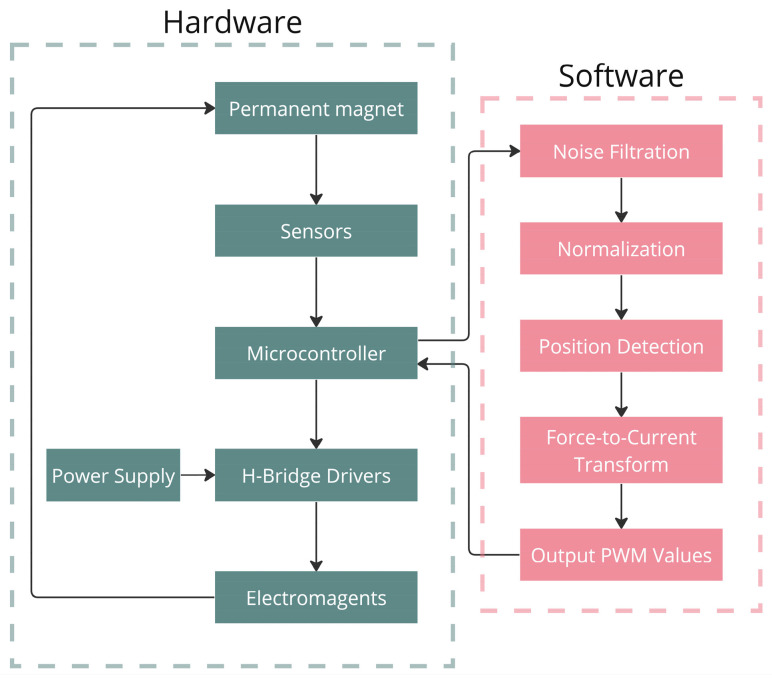
Operational flow of the proposed system.

The system operates as a feedback loop. It consists of an array of electromagnets that balance a permanent magnet above them. A microcontroller adjusts the current flowing through the electromagnets to maintain the magnet’s stable position by continuously responding to its movements. Figure 1 presents a flow chart that outlines the operation processes from both hardware and software perspectives.

The system operation flow begins with the permanent magnet whose behavior determines how the rest of the system will react. The magnetic fields it generates are detected by sensors, which send their values to the microcontroller, where the software process begins. The processing starts by filtering noise from the input signals, then normalizing the values. This is needed because the neural network (NN) that predicts the magnet’s position is trained on normalized data for improved training performance [24], so sensor values must be scaled accordingly for accuracy.

Inference is run continuously, and once the prediction is made, the forces that correspond to the predicted position (pre-calculated via a simulation tool) are retrieved from a lookup table. These forces are used to construct a matrix that converts user-defined forces (e.g., to overcome gravity) into current values. The current values are then scaled into a range to fit the pulse width modulation (PWM) commands. PWM is the method used to control the power delivered through the current drivers drawn from the power supply. Using the PWM values, the microcontroller controls the H-Bridge drivers, adjusting the current to the electromagnets, allowing precise control of the magnetic fields.

**Figure 2:**
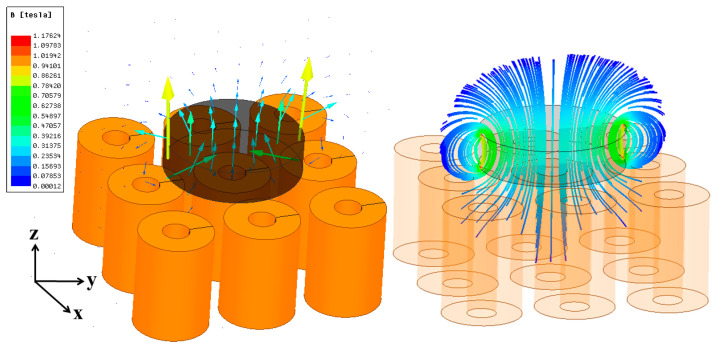
Simulation of a 9-coil array levitating a permanent magnet. The magnetic flux density (in Tesla, T) is visualized using two representations: (**left**) arrow plot, (**right**) streamline plot.

Before proceeding with the hardware design, key system parameters—such as the minimum number of coils, inter-coil spacing, coil turns, input current, and lift-up distance—were investigated and optimized to determine the required levitation force for the permanent magnet. The proposed design was modeled in Ansys Maxwell, and the system’s magnetic behavior was simulated using finite-element analysis (FEA). First, the force required to counteract gravity along the *Z*-axis was calculated based on the permanent magnet’s weight. Next, the optimal inter-coil distance was determined by balancing effective lift distance against translational range. Excessive spacing reduces lifting force, while insufficient spacing restricts the magnet’s movement. Figure 2 illustrates a levitation system comprising a 9-coil array and a cylindrical permanent magnet. The magnetic flux density was analyzed to identify parameters capable of generating sufficient force to levitate the magnet 10 mm above the array. The arrow plot illustrates the vector direction and magnitude of the flux density, while the streamline plot visualizes the field lines.

## 3. Hardware Design

**Figure 3:**
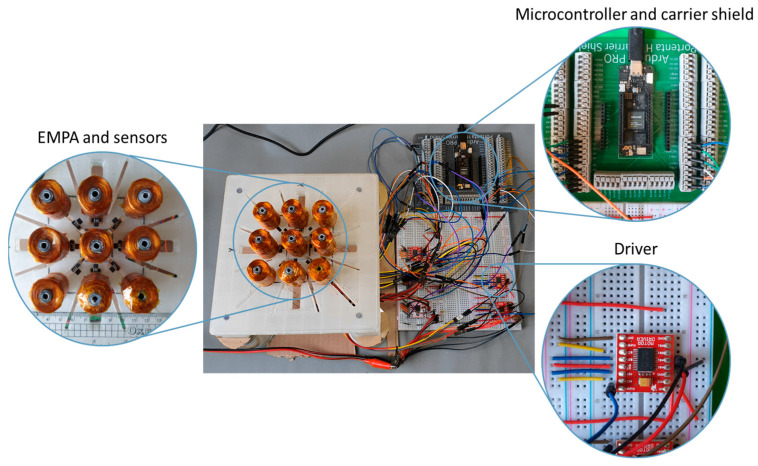
System architecture diagram showing the three main hardware sections.

The system architecture, illustrated in Figure 3, is built around an Arduino Portenta H7 microcontroller, chosen for its low-latency operation, AI capabilities, and compatibility with the Arduino ecosystem. This dual-core board integrates an Arm Cortex-M7 (480 MHz) and an M4 (240 MHz) processor, enabling the concurrent execution of TensorFlow Lite-based machine learning algorithms. To expand its I/O capabilities, an extension board was incorporated using Florent Giraud’s carrier board [25] due to the unavailability of the official Arduino hardware. This setup provides 10 PWM outputs and 8 high-precision (16-bit) ADC inputs, allowing independent control of 10 coils and real-time data acquisition from 8 sensors. Given these hardware constraints, the prototype implements a 3 × 3 square array of 9 coils to validate system functionality while maintaining hardware feasibility. Power management was addressed through a TB6612FNG motor driver IC, which supplements the microcontroller’s limited current output by delivering 1.2 A per channel to two electromagnets. Operating at 2.7–5.5 V logic voltage, the driver enables PWM-based current adjustment for precise levitation force control. Magnetic field measurement employs Texas Instruments’ DRV5055-Q1 Hall-effect sensor (50 mV/mT sensitivity, ±3 mV/mT precision), chosen for its 3 V compatibility with the Portenta. The levitation system uses a First4Magnets N42 neodymium magnet (40 mm diameter, 10 mm height, 1.1–1.3 T remanence) to minimize required coil currents. It is worth emphasizing that while the proof-of-concept implementation was demonstrated on the hardware specified above, it is not fundamentally tied to these components.

The system incorporates a custom-designed adjustable coil stand, as shown in Figure 3, created using Autodesk Inventor, to ensure precise component positioning. Post-assembly validation tests demonstrated excellent agreement with simulation results: at a current of 1 A, the coils achieved the target 10 mm magnet displacement, precisely matching the FEA predictions.

## 4. Software Development

The software architecture integrates two computationally intensive components working in tandem: a machine learning-based position detection system that locates the permanent magnet, and a matrix transformation module that calculates the required levitation currents. Together, these elements enable precise real-time control of the levitating object.

### 4.1. Position Detection

**Figure 4:**
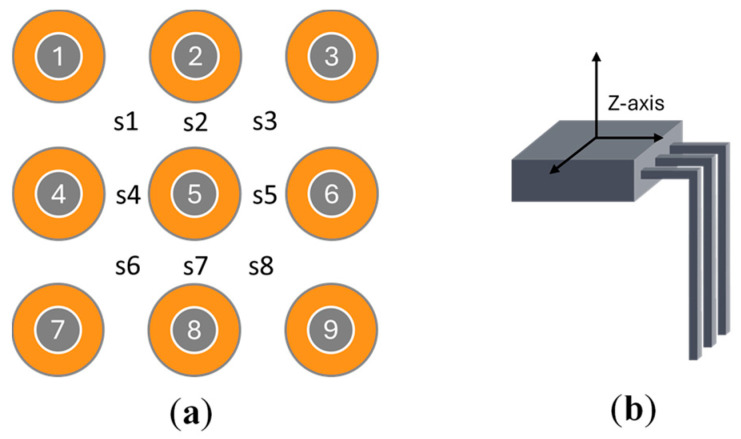
Sensor placement. (**a**) Coil and sensor positions. (**b**) Sensor orientation.

The system utilizes an array of Hall-effect sensors strategically positioned among the coils, as illustrated in Figure 4, to detect magnetic fields along a single axis. Each sensor is oriented with its like-polarity side (defined as the “Top” in the datasheet) facing upward toward the permanent magnet, achieved by bending the sensor legs at 90 degrees.

**Figure 5:**
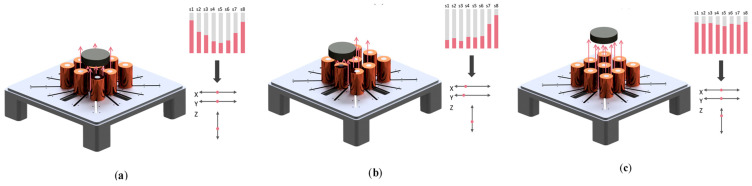
Sensor value variations at different permanent magnet positions. (**a**) Position 1. (**b**) Position 2. (**c**) Position 3.

This configuration allows detection of magnetic fields from multiple directions at varying intensities. As the levitated object moves, each sensor generates a distinct signal, creating a unique pattern of readings, as shown in Figure 5. A dedicated position detection program translates these analog measurements into three-dimensional coordinates (*X, Y, Z*) through nonlinear transformations defined by (1)–(3), where functions *f*, *g*, and *h* represent the mathematical relationships between sensor outputs and the object’s spatial position.

(1) X=f(s1, s2, s3, s4, s5, s6, s7, s8)

(2) Y=g(s1, s2, s3, s4, s5, s6, s7, s8)

(3) Z=h(s1, s2, s3, s4, s5, s6, s7, s8)

The system employs a neural network to approximate these equations, leveraging their known capability for complex function approximation. The trained neural network then translates sensor readings into three-dimensional coordinates by modeling the underlying mathematical relationships.

While individual Hall-effect sensors are limited to measuring magnetic fields along a single axis, combining the outputs from multiple sensors enables accurate estimation of an object’s position. This principle is closely related to sensor fusion, in which data from different types of sensors—such as accelerometers, gyroscopes, and magnetometers—are integrated to estimate orientation parameters like pitch, roll, and yaw [26]. On their own, none of these sensors can provide a complete picture, but their combined measurements yield a robust and reliable estimate. A comparable concept has also been applied in recent research on battery health prediction, where the TinyML framework fuses multiple sensor signals to estimate remaining useful life [27].

For model development, a comprehensive dataset containing sensor values paired with their corresponding position coordinates was created. This dataset enabled supervised training of a neural network to predict the levitated object’s position from unseen sensor data. The resulting model effectively approximates (1)–(3), providing real-time position estimation of the permanent magnet.

#### 4.1.1. Creating the Dataset

The dataset was obtained experimentally by systematically recording sensor values at predefined positions across three axes (X, Y, Z) and two orientations (pitch and roll). The X and Y axes spanned from −20 mm to 20 mm in 1 mm increments, while the *Z*-axis covered 0 mm to 15 mm with the same increment. A guiding coordinate system, fabricated from plastic and cardboard with printed coordinate markings on its base (X-Y) and legs (Z), facilitated precise positioning of the magnetic body. Hall-effect sensors, permanently affixed in place, measured the field at each predefined coordinate.

Measurements were acquired by sequentially positioning the permanent magnet at each target location, capturing data, and then moving to the next coordinate. This process yielded 113 unique spatial positions. To account for orientation variations, pitch and roll angles were introduced, ranging up to 10 degrees in 5-degree increments, expanding the dataset to 365 unique position-orientation combinations. Orientations were only considered up to 10 degrees as a complete rotation of the permanent magnet would be physically impractical, even if the detection algorithm supported it, as the hardware lacks the necessary actuation power and the coils cannot generate the required currents.

To improve robustness against noise and environmental fluctuations, multiple recordings were taken per combination, amassing 13,836 initial data points. A trimmed dataset (5545 points) was subsequently created by removing redundant measurements, mitigating overfitting risks while maintaining a comprehensive spatial and angular coverage.

Notably, measurements were conducted with the coils deactivated, as preliminary tests confirmed their negligible influence on sensor readings. The Hall-effect sensors, positioned 15 mm from the coils and positioned at the electromagnets’ midpoint (where the induced magnetic field is weakest), exhibited no detectable response even at 1 A coil current. This was also theoretically validated by calculating the produced magnetic fields by the coils at the sensor locations which were at or below the sensitivity range of the sensors. To further enhance signal integrity, a Kalman filter [28] was implemented to suppress noise and improve measurement accuracy.

#### 4.1.2. Neural Network

The neural network was implemented in Python 3.11 using TensorFlow 2.11 [29] and developed on a local Windows machine. The methodology described applies uniformly to all models tested in this study.

The training process commenced with dataset normalization [24], where sensor values were scaled using the dataset’s mean and standard deviation to improve model convergence such as:

(4) $$x^{\prime }=\;\frac{x-\mu }{\sigma }$$

where $x^{\prime }$ are the normalized values, *x* are the original values, μ is the mean of the input dataset and σ is the standard deviation.

The normalized data was then partitioned into features (sensor readings) and target variables (position coordinates), followed by a strategic 60-20-20 split into training, validation, and test sets based on established methodology [30].

The implemented neural network architecture employs a multi-output regression approach [31] to predict continuous 3D coordinates. Designed as a sequential model with dense inter-layer connectivity, the network processes inputs through a stacked series of transformations. Two optimal architectures emerged from the experimentation: Type-1 (30-80-60-80-30 hidden layers) with 15,013 parameters and Type-2 (60-80-80-60-30) with 18,683 parameters. Both configurations feature an 8-neuron input layer corresponding to the sensor array and a 3-neuron output layer representing spatial coordinates.

Neuron activations follow the mathematical formulation in (5), where output depends on weighted inputs, bias terms, and the chosen activation function [32,33].

(5) $$a_{i}=\phi (\sum_{j=1}^{k}w_{ij}\;x_{j}+b_{i})$$

where $a_{i}$ is the activation of the ith neuron, ϕ is the activation function, $w_{ij}$ denotes the weight between the ith neuron and the jth input, $x_{j}$ is the jth input to the neuron, $b_{i-}$ is the bias associated with the ith neuron and *k* is the number of inputs to the neuron.

During training, an optimization algorithm iteratively adjusts the network’s weights and biases by minimizing prediction error through backpropagation, with forward propagation computed via (6). The selection and impact of specific activation functions are analyzed in detail in Section 5 (Results and Discussion).

(6) $$h^{\left(l\right)}=\phi (W^{\left(l\right)}*h^{\left(l-1\right)}+b^{l})$$

Here, $h^{\left(l\right)}$ represents the activation at the nth layer, ϕ is the activation function, $W^{\left(l\right)}$ denotes the weight matrix for the nth layer, $h^{\left(l-1\right)}$ is the activation from the previous layer and $b^{\left(l\right)}$ is the bias vector for the nth layer. This process iteratively adjusts the weights and biases from the input layer towards the output layer, optimizing the model’s performance.

Finally, the output layer computes the coordinates [X, Y, Z] without an activation function shown by (7) where *L* is the final layer:

(7) $$[X,\;Y,\;Z]\;=\;W^{L}*h^{L-1}+b^{L}$$

To measure how far the network’s predictions are from the actual values, the mean squared error (MSE) loss function [34] was used as a metric. The MSE is calculated by (8) [35] where *n* is the number of datapoints, $y_{i}$ is the true value and ${\hat{y}}_{i}$ is the predicted value.

(8) $$MSE=\frac{1}{n}\sum_{i=1}^{n}{\left(y_{i}-{\hat{y}}_{i}\right)}^{2}$$

As a second validation measure, the mean absolute error (MAE) function was added to help further assess the performance of the model. The MAE is calculated using (9) [36] where *n* is the number of datapoints, $y_{i}$ is the true value and ${\hat{y}}_{i}$ is the prediction.

(9) $$MAE=\frac{\sum_{i=1}^{n}\left|{\hat{y}}_{i}-y_{i}\right|}{n}$$

#### 4.1.3. Training and Testing

The model training was performed on an Intel Core i7-9750H CPU without GPU acceleration. Initial training parameters were set to 3000 epochs with a batch size of 32, while early stopping mechanisms with patience parameters of 50, 150, 200, and 500 epochs were implemented to prevent overfitting and optimize computational efficiency. These measures effectively limited training sessions to under 10 min due to the model’s compact architecture and efficient stopping criteria. Modern CPUs and GPU acceleration can train these models in under a minute.

**Figure 6:**
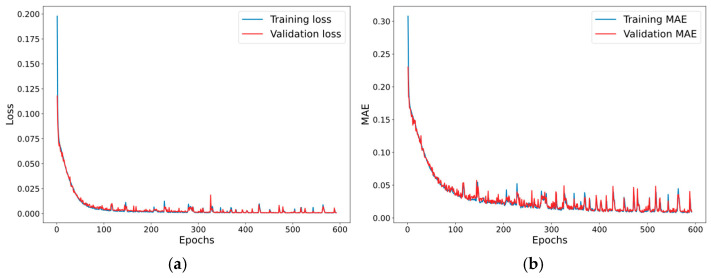
Training metrics for model {Type-1, TanH, NAdam} with batch size of 32. (**a**) Loss (MSE) vs. epochs. (**b**) Mean absolute error vs. epochs.

An extensive evaluation of over 300 model configurations was conducted, examining various activation functions (ReLu, SeLu, TanH, Sigmoid, SoftMax, swish, HardSigmoid, GeLu, and Elu [37]) combined with multiple optimizers (RMSprop, Adam, Nadam, Adamax, Adagrad, and SGD [38,39]). Models achieving MAE below 0.1 mm were selected for conversion to both standard and quantized TFLite formats, enabling microcontroller deployment and performance benchmarking. The comparative analysis revealed superior performance from non-quantized models using the Adam optimizer with TanH activation (see Figure 6 for metrics), with detailed results presented in the dedicated results section.

**Figure 7:**
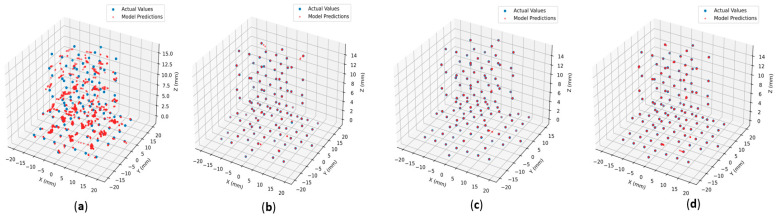
Model predictions against actual values. (**a**) Poorly performing model trained on the extended dataset {Type-2, GeLu, Adagrad}. (**b**) Well-performing model trained on extended dataset {Type-2, TanH, Adamax}. (**c**) Well-performing model trained on the trimmed dataset {Type-2, TanH, Nadam}. (**d**) Well-performing model trained on the trimmed dataset {Type-1, TanH, Nadam}.

Model evaluation focused on loss metric comparisons between training and validation datasets. The consistent pattern of validation metrics slightly outperforming training metrics indicated neither underfitting nor overfitting. As demonstrated in Figure 7b–d, the model maintained strong predictive accuracy on unseen test data, even with trimmed datasets excluding repeated values. However, Figure 7a illustrates how performance variability could occur depending on the specific activation function and optimizer combination, highlighting the importance of careful parameter selection.

#### 4.1.4. Optimizing for Low-Powered Devices

While TensorFlow provides robust machine learning capabilities, its direct deployment on resource-constrained platforms like microcontrollers requires conversion to TensorFlow Lite (TFLite). This format offers enhanced efficiency and supports optimization techniques such as quantization, which reduces numerical precision from 32-bit floating points to 8-bit integers. The quantization process yielded a fourfold reduction in model size while maintaining acceptable accuracy, significantly improving computational performance on embedded systems.

For microcontroller deployment, the optimized TFLite model was converted to a C + + implementation. This conversion involved embedding the neural network architecture along with normalization parameters (mean and standard deviation) into Arduino-compatible code. The implementation also incorporated preprocessing routines for sensor data handling, including analog input filtering and global variable initialization.

**Figure 8:**
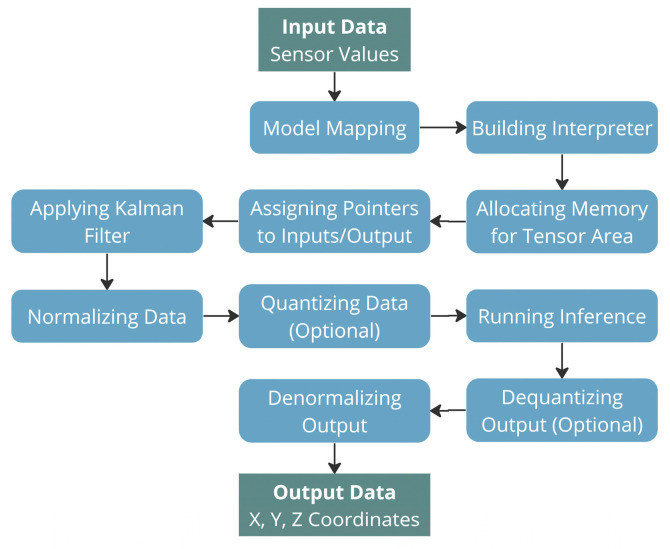
Position detection program flow.

Figure 8 illustrates the software architecture, starting with model initialization in the setup function. This initialization phase handles memory allocation for the model, configuration of the TFLite interpreter, and setup of input/output tensor pointers. During runtime execution, the main processing loop follows a sequential workflow.

First acquiring and filtering sensor inputs, then normalizing the data before performing inference with the quantized model, and finally dequantizing and denormalizing the output tensors to derive the final position coordinates (X, Y, Z). While quantization delivers substantial efficiency improvements, the system maintains the capability to run full-precision 32-bit models when sufficient computational resources are available.

### 4.2. Force-to-Current Matrix Transformation

The control system’s primary objective is to determine the coil currents necessary to generate precise forces and torques for stable magnetic levitation. To meet the 500–1000 Hz operational requirement for the feedback control loop established in prior literature [13,15,16,17], a force-to-current transformation algorithm was developed and validated. The algorithm computes the required coil currents by modeling the electromagnetic interactions between the coil array and levitated object. Since the forces and torques exerted by each coil depend on its current and the object’s known position, these relationships can be expressed through a matrix transformation. Matrix inversion then yields the necessary currents to produce the desired net force and torque on the permanent magnet, as documented in [13,16] and detailed in Algorithm 1.

For development and verification, the algorithm was first implemented in MATLAB R2023b for simulation and analysis. Following validation, it was translated into optimized C + + code for real-time operation. The complete control system, integrating both the position detection and current calculation algorithms, was deployed on an Arduino platform to achieve closed-loop magnetic levitation control.
**Algorithm 1** Force-to-Current Transform
**Input:** Magnet’s *X*, *Y*, *Z* position, desired forces $F_{x}$, $F_{y}$, $F_{z}$, and torques $T_{x}$, $T_{y}$.
**Output:** Vector *I* holds the coil currents required for stable levitation.
**Procedure:**
**1.**Determine the permanent magnet’s position.
**2.**Initialize values for the desired forces ($F_{x}$, $F_{y}$, $F_{z}$) and torques ($T_{x}$, $T_{y}$).
**3.****for** each coil **do**:


1. Calculate radial distance $r_{n}$ and direction $\theta_{n}$.


2. Estimate the force and torque components $\left[f_{x}\left(r_{n},\;z_{n}\right),\ldots \;\tau_{y}\left(r_{n},\;z_{n}\right)\right]$ at the point of interest based on the simulated lookup table using interpolation and extrapolation techniques.


3. Construct matrix *A* from the rotation matrix and the estimated force and torque values.


4. Compute $\;A^{+}$, the pseudoinverse of matrix *A*.


5. Calculate the required current vector $I=A^{†}F$ by setting *F* to be the desired forces $F=[F_{x}$, $F_{y}$, $F_{z}$, $T_{x}$, $T_{y}]$ to achieve levitation.

**end**

## 5. Results and Discussion

In this investigation, over 300 models were explored, varying in architecture, optimizer, activation function, batch size, and patience threshold. The complete dataset and source code for our implementation are publicly available at https://github.com/zal-nemeth/acels (accessed on 15 October 2025). Models that achieved an MAE of 0.1 mm or lower were converted to TensorFlow Lite (TFLite) and their quantized versions were generated for hardware deployment. These models, along with their quantized counterparts, were further transformed into C++ for hardware integration tests using unseen input data.

### 5.1. Integration Results

Benchmarking focused on four key metrics—MAE, MSE, RMSE, and inference runtime—across both quantized and non-quantized models. Table 1 summarizes the results obtained when implementing the position detection model on hardware without additional features such as the matrix transform.

Although quantization predictably resulted in a decrease in accuracy, it did not uniformly enhance runtime efficiency; in certain cases, runtime increased substantially, notably for models utilizing the Nadam optimizer and TanH activation function. Based on this analysis, the top ten models in terms of accuracy and runtime performance were identified. These superior models demonstrated an MAE between 0.026 and 0.038 mm, MSE ranging from 0.011 to 0.060 mm^2^, RMSE between 0.10 and 0.25 mm, and average inference times between 583 and 695 microseconds. These models were subsequently integrated with the matrix transformation algorithm and a PID control placeholder to facilitate the full control system implementation.

Considering the observed accuracy reduction associated with quantization, the non-quantized models were selected for complete hardware deployment. The integrated system exhibited an average runtime between 992 and 1169 µs per control cycle, corresponding to a sampling rate of approximately 855 to 1008 Hz. This is within the operational range required for stable levitation. This runtime encompasses neural network-based position detection and the subsequent force-to-current transformation essential for levitation control. The individual model runtimes are detailed in Table 2.

While the current implementation includes only a basic, non-tuned PID controller, it was incorporated to enable comprehensive timing analysis, as the runtime measurements are independent of controller tuning and thus accurately reflect the achievable loop frequency.

The variation in timing between the models was solely due to the difference in architecture. However, Table 2 presents only the average values, whereas the timings actually ranged between 800 and 1600 µs. The slower speeds occurred at the edge of the detection boundary, where some of the models had more difficulty approximating the position of the object as they reached locations that were less represented in the dataset. Slower response times also occurred when the sensor values changed rapidly. The highest response times observed were below 1600 µs which equates to roughly 625 Hz. This means that even during the slowest performance conditions the system can respond fast enough to remain above the required 500 Hz threshold.

### 5.2. Performance Comparison with Related Work

A detailed comparison with existing approaches is provided in Table 3, where the system’s MAE is listed as “Accuracy” to facilitate direct comparison with metrics used in other solutions. The table compares sensors and control hardware from various studies, alongside key performance metrics such as accuracy and sampling rate. Despite its compact and efficient design, our system achieves an accuracy and sampling rate comparable to those of established solutions, some of which utilize high-end, commercially available tracking systems. Although slightly lower in accuracy, our approach remains competitive and offers a cost-effective alternative. The proposed design offers several notable advantages, including accessibility, low implementation costs, low power consumption and scalability potential. To illustrate the efficiency of the proposed system, an estimate of its power and energy consumption was carried out. Considering only the microcontroller and (3.3 V supply, eight sensors at 48 mA, and the Portenta’s M7 board at 230 mA), the worst-case power consumption is approximately 0.917 W. For a detection cycle of 1 ms, this corresponds to an energy usage of around 917 µJ per cycle.

Additionally, it is a fully standalone system, with the entire control logic implemented on a single chip, eliminating the need for continuous PC connection and enabling portability after programming. The levitated object requires no wires or external batteries, further reducing hardware costs and simplifying deployment.

### 5.3. Limitations and Potential Improvements

Despite demonstrating robust model performance, it is important to acknowledge inherent limitations of the proposed design.

The use of Hall-effect sensors introduces inherent constraints, the most significant being their need for close proximity to the magnetic object. In many applications this requirement is acceptable, but in cases where the object must be detected at a distance, this magnetic sensing approach cannot serve as a substitute for optical methods.

Another limitation lies in the achievable degree of control. While the proposed method provides reliable position detection, it only enables six-degree-of-freedom manipulation of the permanent magnet in a small range. This shortcoming is not due to sensing alone but rather to the system as a whole: the electromagnetic coils are unable to generate sufficient torque to rotate the magnet freely beyond a certain threshold. Limited rotational control is possible; however, as the magnet approaches 90°, stability deteriorates markedly under the current coil design. Within these boundaries, the method remains well-suited for position tracking but cannot offer full unrestricted orientation control.

In terms of accuracy, the main limiting factors are the size and architecture of the neural model and the quantity of training data. In this study, the accuracy converged in the range of 0.0263–0.0381 mm. While the training dataset could be extended without difficulty, the model architecture involves a trade-off: a lightweight model ensures fast predictions but may compromise generalization and accuracy, whereas a larger model would improve precision at the expense of speed. Given that the permanent magnet under investigation is relatively large (40 mm in diameter), we prioritized prediction speed over maximizing accuracy, since sub-40 µm precision was deemed sufficient. The accuracy and generalizability of the models depend heavily on the dataset used for training. Alterations to the physical layout of the system necessitate the collection of new data, as existing models cannot adapt to changes without retraining—a process that can be time-consuming. However, analogous calibration requirements are common across position detection systems. To mitigate this, reinforcement learning strategies could be employed to enable real-time adaptation to dynamic environments.

Future enhancements could focus on improving both inference speed and accuracy by enlarging and diversifying the training dataset, either through additional experimental measurements or by applying Sim-to-Real techniques from robotics [40] for initial training in virtual environments. Further improvements may come from exploring more advanced yet computationally efficient architectures, such as compact convolutional or attention-based networks, implementing orientation predictions, distributing computational tasks across multiple microcontrollers for parallel processing, developing FPGA-based hardware for position detection, and employing adaptive calibration methods to better handle edge-of-volume conditions.

## 6. Conclusions

This paper presented an electromagnetic levitation system built upon a grid-like electromagnetic array with individually controllable coils. To address the high costs associated with traditional position detection methods, a cost-effective alternative was proposed, utilizing inexpensive Hall-effect sensors combined with supervised machine learning techniques. Experimental results confirmed that the system can be effectively implemented on low-power microcontrollers, delivering accuracy and response times comparable to existing solutions, both of which are sufficient to maintain stable levitation. This advancement not only demonstrates the feasibility of deploying comprehensive control algorithms on resource-constrained hardware but also underscores the potential of integrating machine learning to enhance the functionality and scalability of small-scale levitation devices. Overall, the findings push the boundaries of the TinyML framework and suggest promising avenues for future development of compact, efficient, and intelligent levitation systems.

## Figures and Tables

**Table 1:** Model Performance.

Model	Architecture	Activation	Optimizer	MAE (mm)	MSE (mm^2^)	RMSE (mm)	Avg. Runtime (µs)
1	Type-1	TanH	Adam	0.0329	0.0105	0.1026	633.04
2	Type-1	TanH	Nadam	0.0295	0.0188	0.1373	583.07
3	Type-2	TanH	Nadam	0.0263	0.0384	0.1960	694.22
4	Type-2	TanH	Adamax	0.0281	0.0354	0.1881	692.00
5	Type-2	Sigmoid	Nadam	0.0308	0.0603	0.2456	626.27
6	Type-2	Swish	Nadam	0.0442	0.0440	0.2098	678.17

**Table 2:** Model Runtimes.

Model	MAE (mm)	Avg. Runtime (µs)
1	0.0329	1034.21
2	0.0295	1017.21
3	0.0263	1169.71
4	0.0281	1043.19
5	0.0308	992.55
6	0.0442	1011.71

**Table 3:** Solution comparison.

Refs	Sensors	Hardware	Accuracy (mm)	Sampling Rate (Hz)	Cost
[15]	LED + Eddy Current	PC	-	500	High
[13]	Optical motion tracking	PC	0.01	500	High
[16]	Optical motion tracking	PC	0.01	860	High
[17]	Optical motion tracking	PC	0.01	1000	High
[19]	Optical motion tracking	PC	0.01	1000	High
[18]	Laser+ Eddy current	Sainsmart microcontroller	0.008	1000	Low
[4]	Eddy Air Gap	FPGA	0.02	1000	-
This work	Hall-effect	Portenta microcontroller	0.026–0.040	855–1008	Low

## Data Availability

The complete data and source code for our software implementation can be downloaded at: https://github.com/zal-nemeth/acels (accessed on 10 September 2025). Further inquiries can be directed to the corresponding author.

## Abbreviations

The following abbreviations are used in this manuscript:

PID	Proportional–Integral–Derivative
Maglev	Magnetic levitation
NN	Neural Network
PWM	Pulse Width Modulation
MAE	Mean Absolute Error
RMSE	Root Mean Squared Error
MSE	Mean Squared Error
FPGA	Field Programmable Gate Array
PC	Personal Computer
EMPA	Electromagnetic Planar Array
TinyML	Tiny Machine Learning
TF	TensorFlow
